# Supplementary material for: Estimating age of mule deer in the field: Can we move beyond broad age categories?
Source: PLoS One. 2023 Jul 28;18(7):e0284565. doi: 10.1371/journal.pone.0284565 (PMC10381091; doi:10.1371/journal.pone.0284565)
Supplement: Attachment — Submitted filename: Response to Reviewers.docx [file pone.0284565.s002.docx]

28 December 2022

Dr. Jorge Ramón López-Olvera

Academic Editor

*PLOS ONE*

em@editorialmanager.com

Dr. López-Olvera,

We are writing to re-submit our revised manuscript, “Estimating age of mule deer in the field: Can we move beyond broad age categories?” for publication in *PLOS ONE.*

We have edited our manuscript according to the suggested revisions by three anonymous reviewers and yourself. Additions/deletions that did not require a response can be found in the “Revised Manuscript with Tracked Changes” version of the manuscript. We appreciate the helpful feedback that was provided and appreciate the opportunity to submit an improved version of the manuscript. We have provided an in-depth summary of how we addressed each comment on the subsequent pages.

Additionally, we have amended our Role of Funder statement, which is included below.

“Funding for this research was provided by the American Society of Mammalogists (https://www.mammalsociety.org/), Brigham Young University (https://www.byu.edu/), the Bureau of Land Management (https://www.blm.gov/), the Mule Deer Foundation (https://muledeer.org/), the Rocky Mountain Elk Foundation (https://www.rmef.org/), Safari Club International (https://safariclub.org/), Sportsmen for Fish and Wildlife (https://sfw.net/), the Utah Archery Association (https://www.utaharchery.org/), and the Utah Division of Wildlife Resources (https://wildlife.utah.gov/). Grant numbers 196313 and 206012 from Utah Division of Wildlife Resources were awarded to BRM and RTL. Additionally, the Utah Division of Wildlife Resources contributed to the data collection associated with this research. All other funders had no role in study design, data collection and analysis, decision to publish, or preparation of the manuscript.”

Thank you for your continued consideration.

Morgan S. Hinton^1^

Brock R. McMillan

Kent R. Hersey

Randy T. Larsen

^1^Department of Plant and Wildlife Sciences, Brigham Young University

4105 Life Sciences Building

Provo, UT 84602, USA

morganshay527@gmail.com

**Response to Reviewers**

**EDITOR COMMENTS:**

1. Please ensure that your manuscript meets PLOS ONE's style requirements, including those for file naming.

-We have reformatted the manuscript and figures to meet PLOS ONE’s style requirements.

2. Thank you for stating the following financial disclosure:

Please state what role the funders took in the study. If the funders had no role, please state: "The funders had no role in study design, data collection and analysis, decision to publish, or preparation of the manuscript."

If this statement is not correct you must amend it as needed.

Please include this amended Role of Funder statement in your cover letter; we will change the online submission form on your behalf.

-We have amended the Role of Funder Statement and included it in the cover letter.

3. Your ethics statement should only appear in the Methods section of your manuscript. If your ethics statement is written in any section besides the Methods, please delete it from any other section

-Ethics statement has been moved to the Methods section and removed from the end of the document.

4. We note that Figure 1 in your submission contain [map/satellite] images which may be copyrighted. All PLOS content is published under the Creative Commons Attribution License (CC BY 4.0), which means that the manuscript, images, and Supporting Information files will be freely available online, and any third party is permitted to access, download, copy, distribute, and use these materials in any way, even commercially, with proper attribution. For these reasons, we cannot publish previously copyrighted maps or satellite images created using proprietary data, such as Google software (Google Maps, Street View, and Earth). For more information, see our copyright guidelines: http://journals.plos.org/plosone/s/licenses-and-copyright.

We require you to either (1) present written permission from the copyright holder to publish these figures specifically under the CC BY 4.0 license, or (2) remove the figures from your submission:

-Figure has been edited to remove copyrighted satellite imagery from map. The regional boundary layer for this figure is owned by the Utah Geospatial Resource Center and can be located at this link <https://utah.maps.arcgis.com/home/item.html?id=543fa1f073714198a3dbf8a292bdf30c>

This layer is licensed under a CC-BY 4.0 License and open for public use, as shown on the page.

**REVIEWER COMMENTS**

Reviewer #1: This is a well-written manuscript with information that contributes to the literature of aging mule deer, which is arguably growing in importance, given mule deer populations range-wide. Just some minor questions and thought-provoking (hopefully) quibbles.

Lines 68-70; 142-144: The suggestion that aging deer (using the tooth eruption and wear technique [TEW]) at 2.5 years of age is just as accurate as aging deer at 1.5 years of age is misleading and, I would argue, false. At 2.5 years of age, mule deer and white-tailed deer have their permanent (adult) teeth and dental formula, with the only distinguishable features between deer 2.5 years and older deer being tooth wear patterns, which can sometimes be difficult to identify all necessary characteristics in the field on a live individual and can vary based on diet, size of deer, and other factors, as you know. Hamlin et al. (2000), Storm et al. (2014), and Adams & Blanchong (2020) all show error using TEW for aging deer 2.5 years and older.

-We are a little confused by this comment because Figure 3 shows decreased accuracy for 2.5 year-old deer compared to 1.5 year-old deer. For both ages, accuracy was high, however, the point estimate for accuracy of 2.5 year olds is lower. Does the comment refer to Figure 4 regarding bias? Language in the introduction and methods sections has been added to provide clarity.

Lines 242-244, Fig. 2: The odds ratios (ORs) graphed in Fig. 2 do not match those reported in Lines 242-244. From the beta-estimates, it appears the ORs reported in Lines 242-244 are correct.

-We are also confused by this comment. The estimates in lines 242-244 are beta estimates on the logit scale as shown in Table 2. Figure 2 reports odds ratios which are easily calculated from the beta estimates. We’ve adjusted the language in the caption for Figure 2 to more clearly articulate that the figure refers to odds ratios. This change should clarify for the reader.

Line 281: I’m uncertain that most folks would interpret 75% of TEW-CA age estimate pairings within 1 year of each other as “high accuracy”. That would imply that, using TEW aging in the field, managers would be assigning an incorrect age to greater than 25% of deer they’re aging 2.5 years and older. Depending on how different age-specific demographic parameters are from year-to-year, the potential for error could be quite large. While CA aging is not perfectly accurate either, as showed by those cited above as well as Asmus & Weckerly (2002) and Veiberg et al. (2020), CA aging is much more accurate than TEW, especially at older age classes. That’s why most recommend that if managers need ages accurate to the year for age-specific demographic parameters, that CA aging is the best bet.

-We agree that CA is the most accurate method for aging ungulates. This method, however, is invasive and impractical for a live animal being released back into the wild (Festa-Bianchet 2002). Following suggestions from reviewer 2, we have added the term “live” to highlight the TRW method being the most commonly used method for live animals.

Some other questions/thoughts: it would be nice to see a table of the raw TEW estimates vs the CA age estimates to see the "unmodeled" accuracy. Did you explore categories other than 3.5 and older (e.g., 2-4 years, 5-7 years, 8 years and older [not an actual suggestion, just an example, more thought would need to be given to appropriate age categories based on demographic parameters) and if those may be more useful to managers? Key to all of this is do demographic parameters for deer change at different ages? That is where age categories of importance could be identified. Maybe the answer to the question in your title is somewhere in between the 2.5 years and older category of old and the single-year category.

-The raw data for ages estimated by TRW compared to estimates derived from CA for each individual are available for this manuscript. We agree that more specific age categories as mentioned could be beneficial to managers than 3.5+, however, such categories are not yet suggested in the literature. However, categories cannot precisely indicate at which ages demographic parameters change for deer. If such categories are to be determined, further research needs to be conducted to indicate the ages at which demography of mule deer changes.

Reviewer #2: This manuscript evaluates the accuracy of tooth wear and replacement patterns for aging mule deer in Utah by comparing it to cementum analysis. Age information is often important for wildlife population management including estimating population demographic rates. I think the manuscript would be more useful if the authors revised it to increase specificity with respect to the background information and objectives, offer more specific detail about the data collected and how they were used in analysis, and provide a more careful discussion of the interpretation and implications of the findings that brings in the vast literature on this topic.

At the moment, the manuscript lacks important detail in numerous places that makes it difficult to evaluate the Results or the relatively strong conclusions/ recommendations provided in the Discussion and Conclusions sections. Further, in the Introduction there are generalizations made about aging using teeth that are not appropriate. These statements should be revised to more specifically speak to the taxa of interest in the study. The section is also under referenced. The Discussion is extremely under-referenced. Finally, the conclusions and recommendations are overly strong and applied too broadly relative to the data presented and without adequate evidence to support them from the literature (references). I provide specific line-by-line comments below.

-Thank you for the comments. Taxa clarity was added both in wording and in references. Additional references were added to both the introduction and discussion.

Line 53-45 – Sex is not easy to determine in the wild for numerous wildlife species. So, being more specific about which taxa you are referring to would be useful.

-Changed to Cervids.

Line 62-63 – Reference needed.

-References added to support the sentence.

Line 66 - Clarify you mean from live animals.

-Changed with reviewer recommendation

Line 67-70 - This citation is for fallow deer. Is it appropriate to make such a broad generalization based on this one reference for one species?

-Additional citations added and language changed to provide clarity.

Line 71-72- Reference needed.

-References added to the sentence.

Line 73-76 - You are talking about cervids here so be specific. Again, this sentence is making a broad generalization when I don't think you mean to.

-Changed to reviewer recommendation, specified cervids.

Line 85-88 - Again, you are not doing this to generalize to all wildlife (or if that is your intent, I think you should not)...you have a particular taxon of focus and you should state that.

-Changed to reviewer recommendation, specified cervids

Line 91 – Clarify what you mean by “field aging” = using tooth eruption and wear patterns. Also, it should be noted that aging based on cementum annuli is not perfect. There is literature about this. So, you should be careful using it as a “true age”.

-Changed with reviewer recommendation to “tooth replacement and wear method” and changed “true age” to cementum age.

Line 133-134 – What is your basis for 200 deer as the cutoff for experienced vs inexperienced? Justify. How many people did you have in each category? What was the distribution of experience in each category? How many agers in total did you end up having in each category?

-We have added wording to this section to justify this using this number as a cutoff.

Line 134-135 - This is an awkward arrangement. Why talk about 1.5 to 2.5 year olds (without describing how) and then talk about the younger animals (fawns) and then go back to the older animals?

-Changed to reviewer recommendation, changed sentence arrangement to follow ages

Line 187-188 - What does within 1 year mean in terms of what your data were? It's not clear what the age categories are for TWR vs cementum that you are using so I can't tell if within 1 year means the same age or something different. Perhaps you could provide a table with more of the actual data rather than moving straight to outputs of regression models so the reader can see for themselves exactly what you were working with.

-We aged each animal to the year for both CA and TWR (age categories = “1.5, 2.5, 3.5. etc.). We’ve adjusted the wording of lines in the data analysis section to be more clear about the meaning of within 1 year.

Line 207-208 - Justify this. Model averaging is not universally agreed upon as an appropriate strategy. Also justify the choice of 5% with a reference.

-References have been added to support the choice of 5%.

Line 208-211- Why did you do this? And what was your criterion for uninformative parameters? I also don't understand what "models occurred with other competing models" means.

-Thanks for the note. We have added a reference and rewritten the sentence to clarify.

Line 222-223 - Your second model was within 2 AIC of your top model. The standard is generally that any model within 2 AIC of the top model is competitive. So, justify why sex is not important.

-Yes. We model averaged given our results showed multiple competing models. The confidence intervals around the beta estimate for sex, however, overlapped zero suggesting it was not as influential as other variables. We’ve adjusted the language to clarify.

Table 1 – Is “1” the intercept only model? If so, make that clear.

-Changed to Intercept Only in the table.

Line 245-247 - Confusing as written. What age is most accurate for males and how do you know?

-Reworded for clarity.

Table 2 - Why would you choose an 85% CI?

-Because model selection uses a “weight of evidence” approach as opposed to an up or down vote based on a p-value, recommendations now suggest using 85% confidence intervals around beta estimates to assess direction and strength of effect sizes (see Arnold 2010).

Fig 2. – I think this figure is mislabeled. Your experience category has error bars above and below an odds ratio of 1 (1 being no association). This does not make sense in terms of being significant. And, capture period is totally below 1 suggesting it is significant - which is not reflected in Table 2. Also, indicate in the legend what the error bars are.

-Thank you for catching this. The figure labels have been corrected to the appropriate ones, which align with the data shown in Table 2. Figure caption has been changed to denote SE bars.

Fig. 3-5 – What are the error bars and envelopes (shaded areas) on these figures?

-Added language to figure legends to indicate SE bars and confidence intervals.

Fig. 5 – How many deer had to be aged for someone to qualify as experienced? Methods says 200. Here 200 of each sex – so 400 total? This is unclear.

-Thank you for catching this. We have corrected this in the figure caption to reflect the correct number of animals.

Line 280-281 - Clarify what your definition of reliable is.

-Thank you for this comment. Language was added to the sentence for clarity.

Paragraph 1 (line 280-291) - I think you need to go into what this level of "reliability" pertains to. What management contexts would the level of accuracy you found be sufficient and in what contexts/questions would more accurate aging be needed? This is important and should be explored more deeply and use the literature.

-Thank you for this comment. The management contexts associated with the accuracy found in this study are reported, with citations, in lines 321-330.

Paragraph 2 (line 296-307) - This all seems really speculative and you've provided no assessment of accuracy based on experience for males so you really can't support your statements about sex based on what you’ve written here. Also, you need references to support the general statements you are making in lines 301-307.

-Paragraph has been rewritten for clarity. References have been added to these lines.

Paragraph 3 (308-316) and 4 (line317-328) – Again, the statements in these paragraphs need references to support them.

-References have been added to these lines.

Line 308 – What does this sentence mean?

-Reworded and changed language for clarity.

Line 326-428 - Your "high" is subjective. Be specific about what you found.

-We agree that the term high is subjective. We removed the word.

Line 334-336 – I think this is a risky recommendation to make for all mule deer.

-Thank you for the comment. We reworded our conclusions to more accurately reflect the usefulness of this method.

Reviewer #3: Hinton et al. presented a ms entitled “Estimating age of mule deer in the field: Can we move beyond broad age categories?” for publication in PLOS ONE. The paper deals with a relevant and interesting topic: age estimation (teeth eruption/wearing) versus age determination (analysis of cementum annuli from teeth) in ungulates. The ms reported the case of mule deer (Odocoileus hemionous; sample size: 384 unique age estimates for 251 individuals, 347 females and 37 males) aging across the State of Utah.

I enjoyed reading this ms and I think that this study is a valuable contribution to improve the knowledge/management of this species.

However, despite the interesting topic and the potential of management implications for the target species, I believe that a revision is required (please, see my comments below).

General comments:

1) A first flaw that I found concerns the absence of an accurate description of the age estimation method. The conclusions of this ms open up the possibility of using age estimation in future analyzes but do not describe how ages were estimated.

The Authors refer to two old studies and a book (Severinghaus 1949, Robinette 1957, Heffelfinger 2006). However, the experience in age estimation resulted an important variable.

I believe this is a fundamental point: the Authors must describe how ages were estimated by expert biologists through the use of detailed descriptions and photographs. Only in this way their findings can be useful to the scientific community.

-Thank you for this comment. We have added a table in the methods section outlining the general dental characteristics associated with each age of mule deer.

2) The sample of males is very low. I suggest to carry out the analyzes separately for the two sexes.

-The results show that sex was not a significant variable in the model selection process, indicating that there is not a significant difference in age estimates for males vs. females.

3) Is the age of these animals estimated using the same criteria for males and females?

-Yes. An additional sentence was added to clarify the same criteria was used to estimate age of both sexes.

4) I believe it is important to compare animals in which age was estimated by tooth eruption stages and those in which it was evaluated by tooth wear. I have no direct experience on this species but for many other ungulates species the estimation of age by eruption is very different from the wear processes. Is the experience important also using eruption stages?

-These are two different processes used to estimate two different age classes of animals. Estimation using tooth eruption is only applicable to deer 2.5 years old and younger and estimation using tooth wear is only applicable to deer 3.5 years old and greater.

5) Another very important issue is related to the use of animals coming from very different areas (21 management units within five administrative regions of the Utah Division of Wildlife Resources). These areas have very different altitudinal ranges, vegetation communities, etc etc…

The Authors should take into account the “data collection area” in their models, not only as a random factor (e.g., altitudinal range, vegetation, …). This analysis could help to understand if the underestimation is the same over the whole large study area or if this is true only for certain areas (for example areas with lower altitudes).

-Analyses were performed to determine the effect of unit of capture and ecoregion on accuracy of age estimates. Results indicated there was no significant influence of either variable on accuracy of estimates. We added a sentence in the methods section to summarize this finding.
