# Supplementary material for: Estimating age of mule deer in the field: Can we move beyond broad age categories?
Source: PLoS One. 2023 Jul 28;18(7):e0284565. doi: 10.1371/journal.pone.0284565 (PMC10381091; doi:10.1371/journal.pone.0284565)
Supplement: Attachment — Submitted filename: Response to Reviewers.docx [file pone.0284565.s003.docx]

30 March 30, 2023

Dr. Jorge Ramón López-Olvera

Academic Editor

*PLOS ONE*

em@editorialmanager.com

Dr. López-Olvera,

We are writing to re-submit our revised manuscript, “Estimating age of mule deer in the field: Can we move beyond broad age categories?” for publication in *PLOS ONE.*

We have edited our manuscript according to the suggested revisions by three anonymous reviewers and yourself. Additions/deletions that did not require a response can be found in the “Revised Manuscript with Tracked Changes” version of the manuscript. Edits from the first revision are noted in yellow whereas edits from the second revision are marked in red. We appreciate the helpful feedback that was provided and appreciate the opportunity to submit an improved version of the manuscript. We have provided an in-depth summary of how we addressed each comment on the subsequent pages.

Thank you for your continued consideration.

Morgan S. Hinton^1^

Brock R. McMillan

Kent R. Hersey

Randy T. Larsen

^1^Department of Plant and Wildlife Sciences, Brigham Young University

4105 Life Sciences Building

Provo, UT 84602, USA

morganshay527@gmail.com

**Response to Reviewers**

**REVIEWER #1:**

Reviewer #1: The manuscript was improved from its previous version with statements that added clarity. However, after further review, I have concerns about the statistical analyses and the subsequent presentation/interpretation of those results.

Line 140: “…to identify deer 1.5 years old from deer older than 1.5 years.”

- Changed to reviewer recommendation.

Table 1: This table is unnecessary as it is described in your citations [24-25,40]. Additionally, it insinuates that it is a straight-forward method, which it’s known that it isn’t, and that the authors point out in Lines 299-301 (“…due to increased variation in tooth wear for older individuals…”). I would just leave it as it is in Line 148 that older deer were aged based on wear patterns.

- This table was added to the manuscript per request of Reviewer #3: “I believe this is a fundamental point: the Authors must describe how ages were estimated by expert biologists through the use of detailed descriptions and photographs. Only in this way their findings can be useful to the scientific community.” Lines 152-154 caution readers “These characteristics are not absolutes, but rather general guidelines that have been supported by frequent feedback determined from cementum analysis.”

Lines 217-220: The sentences beginning on Line 217 and Line 219 effectively say the same thing. Combine the two.

- Thank you for the comment. Sentences were combined as the reviewer suggested.

Lines 219-220: Although I don’t agree with the model-averaging technique used, if the statement of “much of the work in natural resources” is to be used, more citations would be necessary.

- Added several more citations of studies using model-averaging to this statement.

Lines 220-221: If the models were assessed for evidence of uninformative parameters, why were they still included them in the final model?

- Thank you for catching this. Models judged to be informative were removed from the final model and noted in the model list as uninformative.

Lines 234-235: Again, if a parameter wasn’t statistically supported in any of the models, why was it still included in the final model?

- Same as reply above, uninformative models were removed from final model.

Lines 252-254: Typically, if there’s a significant interaction term included in a model, the two coefficients (Observer & Age, in this case) are nearly meaningless by themselves, as they would be dependent on the other. However, since the interaction term itself is nearly 0 and insignificant, I suppose these are fine. Although I’d again question the inclusion of these terms that are nearly 0. We strive to find the simplest model that explains the most about our data (i.e., the basis of AIC), so why include all these uninformative parameters when two models with 3 different predictor variables were found that best describe the data?

- Same as reply above, uninformative models were removed from final model.

Fig. 2: Why is the interaction term not included when all other variables are?

- Interaction term added to this figure. Thank you for the comment.

Figs. 3 & 5: One of these is unnecessary. Going back to the comments about Lines 252-254: typically, Fig. 3 would not be warranted because the plot of the interaction would illustrate the two variables. But since the interaction is uninformative, the interaction plot isn’t needed either.

- Thank you for this feedback. Removed Figure 5 from manuscript, as the age and observer interaction was found to be uninformative.

Throughout the paper: Choose and stick with one of the commonly used phrases of the “tooth eruption and wear method”, as it is inconsistent throughout (e.g., TRW, TEW, etc.). Line 315 uses “dental wear and eruption method” and Lines 340-341 use “tooth wear and replacement” method, for example.

- Thank you for catching this. All terminology for this method has been changed to “tooth replacement and wear method” to create consistency and align with the most commonly used term for the method.
